# *N*-(2,6-Diisopropyl­phen­yl)formamide

**DOI:** 10.1107/S1600536808024811

**Published:** 2008-08-09

**Authors:** Jackson M. Chitanda, J. Wilson Quail, Stephen R. Foley

**Affiliations:** a Department of Chemistry, University of Saskatchewan, 110 Science Place, Saskatoon, Saskatchewan, Canada S7N 5C9; b Saskatchewan Structural Sciences Centre, University of Saskatchewan, 110 Science Place, Saskatoon, Saskatchewan, Canada S7N 5C9

## Abstract

The title compound, C_13_H_19_NO, exhibits a non-planar structure in which the 2,6-diisopropyl­phenyl ring is tilted at a dihedral angle of 77.4 (1)° with respect to the formamide group. This is the largest dihedral angle known among structurally characterized formamides. The mol­ecules are linked *via* N—H⋯O hydrogen bonds, forming infinite chains which run along the *b*-axis directions.

## Related literature

For related literature, see: Boeyens *et al.* (1988 ▶); Ferguson *et al.* (1998 ▶); Gowda *et al.* (2000 ▶); Krishnamurthy (1982 ▶); LaPlanche & Rogers (1964 ▶); Omondi *et al.* (2005 ▶); Cerecetto *et al.* (2004 ▶); Chitanda *et al.* (2008 ▶).
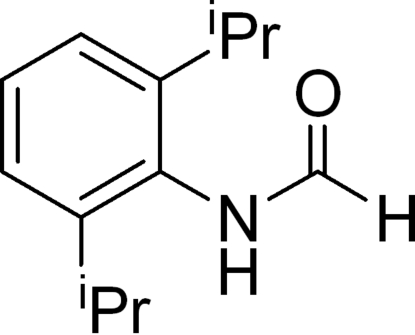


## Experimental

### 

#### Crystal data

C_13_H_19_NO
*M* *_r_* = 205.29
Monoclinic, 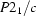

*a* = 8.9581 (15) Å
*b* = 8.7684 (15) Å
*c* = 15.840 (6) Å
β = 105.381 (10)°
*V* = 1199.6 (5) Å^3^
*Z* = 4
Mo *K*α radiation
μ = 0.07 mm^−1^
*T* = 173 (2) K
0.25 × 0.05 × 0.05 mm

#### Data collection

Nonius KappaCCD diffractometer
Absorption correction: none
7758 measured reflections
2365 independent reflections
1556 reflections with *I* > 2σ(*I*)
*R*_int_ = 0.070

#### Refinement

*R*[*F* ^2^ > 2σ(*F* ^2^)] = 0.054
*wR*(*F* ^2^) = 0.128
*S* = 1.05
2365 reflections
140 parameters
H-atom parameters constrained
Δρ_max_ = 0.15 e Å^−3^
Δρ_min_ = −0.20 e Å^−3^

### 

Data collection: *COLLECT* (Nonius, 1998 ▶); cell refinement: *SCALEPACK* (Otwinowski & Minor, 1997 ▶); data reduction: *DENZO* (Otwinowski & Minor, 1997 ▶) and *SCALEPACK*; program(s) used to solve structure: *SIR97* (Altomare *et al.*, 1999 ▶); program(s) used to refine structure: *SHELXL97* (Sheldrick, 2008 ▶); molecular graphics: *ORTEP-3 for Windows* (Farrugia, 1997 ▶); software used to prepare material for publication: *SHELXL97*.

## Supplementary Material

Crystal structure: contains datablocks I, global. DOI: 10.1107/S1600536808024811/bv2100sup1.cif

Structure factors: contains datablocks I. DOI: 10.1107/S1600536808024811/bv2100Isup2.hkl

Additional supplementary materials:  crystallographic information; 3D view; checkCIF report

## Figures and Tables

**Table 1:** Hydrogen-bond geometry (Å, °)

*D*—H⋯*A*	*D*—H	H⋯*A*	*D*⋯*A*	*D*—H⋯*A*
N1—H1⋯O1^i^	0.88	2.04	2.910 (2)	171

Symmetry code: (i) 
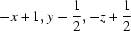
.

**Fig. 1.**
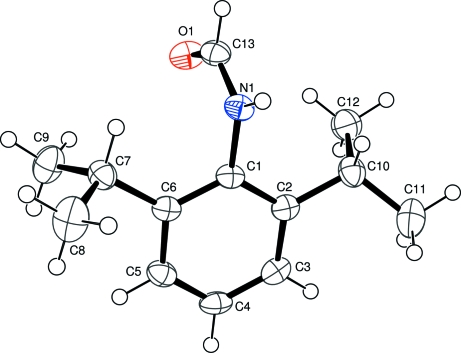
The molecular structure of (I), showing the atom-labeling scheme. Thermal ellipsoids are drawn at the 50% probability level.

**Fig. 2.**
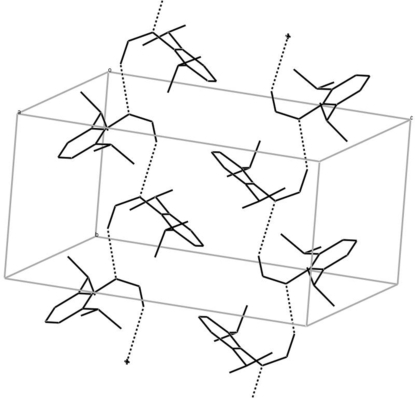
The packing of (I), with hydrogen bonds shown as dashed lines. For clarity, H-atoms have been omitted.

**Fig. 3.**
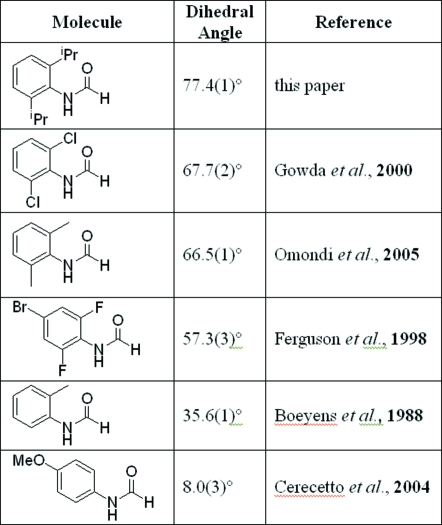
Dihedral angle of previously characterized aryl-substituted formamides

## supplementary crystallographic information

### Comment

As part of the ongoing research in our laboratory directed at the synthesis substituted iminoisoindolines (Chitanda *et al.*, 2008), the title compound was obtained as a by-product and then purposefully synthesized in 92% yield. *N*-(2,6-diisopropylphenyl)formamide has been previously reported (Krishnamurthy, 1982), however no X-ray structure nor NMR data has been previously published. We have now determined the single-crystal X-ray structure of the title compound, (I).

The ^1^H NMR (CDCl_3_) spectra of I is a mixture of two carbon-nitrogen bond rotomers, where the ratio of the major rotomer to the minor rotomer is about 2:1. Upon crystallization however, the solid state structure shows exclusive formation of the cisoidal rotomer. As shown in Figure 1, the carbonyl group on the formamide moiety is positioned almost perpendicular to the plane of the aromatic ring, and is oriented *cis* to the aromatic group about the carbon-nitrogen bond. The dihedral angle between the plane of the aromatic ring and that formed by the N—C=O moiety is 77.4 (1)°, which is considerably larger than the corresponding angle in previously structurally characterized aryl-substituted formamides (Figure 3). This is attributed to the presence of the bulky isopropyl groups on the *ortho* positions of the phenyl ring which increases torsional strain between the two planes defining the dihedral angle. For example, in the less bulky analogue, *N*-(4-methoxyphenyl)formamide, the dihedral angle is only 8.0 (3)° (Figure 3, Cerecetto *et al.*, 2004). The two isomers of the title compound arise due to hindered rotation about the amidic bond (LaPlanche *et al.*, 1964). (I) crystallizes in the monoclinic space group P21/c. The molecules of (I) are linked to form infinite chains which run along the *b* axis direction *via* N—H···O hydrogen bonds (details in Table 3).

### Experimental

The refined procedure for the synthesis of (I) is as follows: A solution of 2,6-diisopropyl aniline (4.695 g, 26.5 mmol s) and formic acid (7.314 g, 159.0 mmol, 6eq.) in chloroform (20 ml) was refluxed with continuous stirring for 16 hrs. The colour of the solution changed from yellow to green to colorless over the course of the reaction. The solvent and excess formic acid were removed under vacuum to yield the title compound as a white solid. Needle-like single crystals suitable for X-ray analysis were obtained from slow evaporation of a chloroform solution (5.00 g, 92%). 1*H*-NMR (CDCl_3_, p.p.m.): Two rotomers observed in 2:1 ratio. Major Rotomer: δ 1.19 (d, J = 6.9 Hz, 12H, –CH(CH3)2), δ 3.08 (septet, J = 6.9 Hz, 2H, –CH(CH_3_)_2_) δ 6.64 (*s*(br), 1H, –NH–), δ 7.17 (m, 2H, aromatic), δ 7.30 (m, 1H, aromatic), δ 8.47 (s, 1H, –C(H)=O). ^13^C-NMR (CDCl_3_, p.p.m.): δ 23.74 (CH(CH3)2), d 28.9 (–CH(CH3)2), d 123.6, δ 128.7, δ 129.9, δ 146.2, δ 161.0 (–C(H)=O). Minor Rotomer: δ 1.20 (d, J = 6.9 Hz, 12H, –CH(CH_3_)_2_), δ 3.20 (septet, J = 6.9 Hz, 2H, –CH(CH_3_)_2_) δ 7.02 (d, J = 11.2 Hz, 1H, –NH–), δ 7.19 (m, 2H, aromatic), δ 7.30 (m, 1H, aromatic), δ 8.0 (d, J = 11.2 Hz, 1H, –C(H)=O). ^13^C-NMR (CDCl_3_, p.p.m.), Major Rotomer: δ 23.77 (–CH(CH_3_)_2_), δ 28.6 (–CH(CH_3_)_2_), δ 123.9, δ 129.0, δ 130.4, δ 146.9, δ 165.9 (–C(H)=O). ESI-MS (m/*z*): calcd. for C13H19NO; 205.1467, 206.1545 [*M*+H]^+^; found; 206.1546 [*M*+H]^+^.

### Refinement

The hydrogen atoms in the ammonium ions in (II) and (IV) were all found in ΔF maps. The hydrogen atoms were placed in calculated tetrahedral positions on the N atoms (N—H = 0.95 Å). The *U*_iso_ of each H atom was assigned as equal to 1.5 times the *U*_eq_ of the attached N atom.

### Crystal data

**Table:**

C_13_H_19_NO	*F*_000_ = 448
*M**_r_* = 205.29	*D*_x_ = 1.137 Mg m^−^^3^
Monoclinic, *P*2_1_/*c*	Mo *K*α radiation λ = 0.71073 Å
Hall symbol: -P 2ybc	Cell parameters from 5165 reflections
*a* = 8.9581 (15) Å	θ = 1.0–27.5º
*b* = 8.7684 (15) Å	µ = 0.07 mm^−^^1^
*c* = 15.840 (6) Å	*T* = 173 (2) K
β = 105.381 (10)º	Rod, colourless
*V* = 1199.6 (5) Å^3^	0.25 × 0.05 × 0.05 mm
*Z* = 4	

### Data collection

**Table:**

Nonius KappaCCD diffractometer	2365 independent reflections
Radiation source: fine-focus sealed tube	1556 reflections with *I* > 2σ(*I*)
Monochromator: horizonally mounted graphite crystal	*R*_int_ = 0.070
Detector resolution: 9 pixels mm^-1^	θ_max_ = 26.0º
*T* = 173(2) K	θ_min_ = 2.4º
φ scans and ω scans with κ offsets	*h* = −11→11
Absorption correction: none	*k* = −10→10
7758 measured reflections	*l* = −17→19

### Refinement

**Table:**

Refinement on *F*^2^	Secondary atom site location: difference Fourier map
Least-squares matrix: full	Hydrogen site location: inferred from neighbouring sites
*R*[*F*^2^ > 2σ(*F*^2^)] = 0.054	H-atom parameters constrained
*wR*(*F*^2^) = 0.128	*w* = 1/[σ^2^(*F*_o_^2^) + (0.0512*P*)^2^ + 0.2338*P*] where *P* = (*F*_o_^2^ + 2*F*_c_^2^)/3
*S* = 1.05	(Δ/σ)_max_ < 0.001
2365 reflections	Δρ_max_ = 0.16 e Å^−^^3^
140 parameters	Δρ_min_ = −0.20 e Å^−^^3^
Primary atom site location: structure-invariant direct methods	Extinction correction: none

### Special details

**Table:**

Geometry. All e.s.d.'s (except the e.s.d. in the dihedral angle between two l.s. planes) are estimated using the full covariance matrix. The cell e.s.d.'s are taken into account individually in the estimation of e.s.d.'s in distances, angles and torsion angles; correlations between e.s.d.'s in cell parameters are only used when they are defined by crystal symmetry. An approximate (isotropic) treatment of cell e.s.d.'s is used for estimating e.s.d.'s involving l.s. planes.
Refinement. Refinement of *F*^2^ against ALL reflections. The weighted *R*-factor *wR* and goodness of fit *S* are based on *F*^2^, conventional *R*-factors *R* are based on *F*, with *F* set to zero for negative *F*^2^. The threshold expression of *F*^2^ > σ(*F*^2^) is used only for calculating *R*-factors(gt) *etc*. and is not relevant to the choice of reflections for refinement. *R*-factors based on *F*^2^ are statistically about twice as large as those based on *F*, and *R*- factors based on ALL data will be even larger.

### Fractional atomic coordinates and isotropic or equivalent isotropic displacement parameters (Å^2^)

**Table:**

	*x*	*y*	*z*	*U*_iso_*/*U*_eq_	
N1	0.51988 (18)	0.05926 (16)	0.22728 (10)	0.0277 (4)	
H1	0.5445	−0.0380	0.2282	0.033*	
O1	0.40304 (16)	0.24069 (14)	0.29095 (9)	0.0385 (4)	
C1	0.5662 (2)	0.15772 (18)	0.16592 (12)	0.0253 (4)	
C2	0.7219 (2)	0.20161 (19)	0.18370 (13)	0.0276 (5)	
C3	0.7655 (2)	0.2939 (2)	0.12239 (14)	0.0311 (5)	
H3	0.8701	0.3260	0.1329	0.037*	
C4	0.6582 (2)	0.3389 (2)	0.04654 (14)	0.0326 (5)	
H4	0.6898	0.4012	0.0053	0.039*	
C5	0.5059 (2)	0.2940 (2)	0.03028 (13)	0.0319 (5)	
H5	0.4337	0.3255	−0.0223	0.038*	
C6	0.4557 (2)	0.20300 (19)	0.08966 (12)	0.0267 (4)	
C7	0.2869 (2)	0.1551 (2)	0.06884 (13)	0.0327 (5)	
H7	0.2744	0.0866	0.1170	0.039*	
C8	0.2398 (3)	0.0654 (3)	−0.01662 (16)	0.0514 (7)	
H8A	0.1320	0.0322	−0.0273	0.062*	
H8B	0.3069	−0.0241	−0.0125	0.062*	
H8C	0.2504	0.1304	−0.0650	0.062*	
C9	0.1804 (2)	0.2920 (2)	0.06525 (17)	0.0460 (6)	
H9A	0.0736	0.2566	0.0564	0.055*	
H9B	0.1867	0.3587	0.0167	0.055*	
H9C	0.2125	0.3487	0.1204	0.055*	
C10	0.8387 (2)	0.1508 (2)	0.26749 (14)	0.0349 (5)	
H10	0.8116	0.0441	0.2800	0.042*	
C11	1.0061 (2)	0.1496 (3)	0.26122 (17)	0.0505 (6)	
H11A	1.0727	0.1026	0.3140	0.061*	
H11B	1.0404	0.2545	0.2560	0.061*	
H11C	1.0125	0.0909	0.2097	0.061*	
C12	0.8260 (3)	0.2501 (3)	0.34482 (15)	0.0468 (6)	
H12A	0.8939	0.2092	0.3991	0.056*	
H12B	0.7187	0.2499	0.3488	0.056*	
H12C	0.8573	0.3548	0.3360	0.056*	
C13	0.4417 (2)	0.1082 (2)	0.28273 (13)	0.0309 (5)	
H13	0.4131	0.0339	0.3192	0.037*	

### Atomic displacement parameters (Å^2^)

**Table:**

	*U*^11^	*U*^22^	*U*^33^	*U*^12^	*U*^13^	*U*^23^
N1	0.0361 (9)	0.0177 (7)	0.0303 (10)	−0.0005 (6)	0.0103 (8)	0.0028 (6)
O1	0.0492 (9)	0.0278 (7)	0.0443 (9)	−0.0025 (6)	0.0228 (7)	−0.0035 (6)
C1	0.0337 (11)	0.0167 (8)	0.0276 (11)	−0.0004 (7)	0.0120 (9)	0.0001 (7)
C2	0.0339 (11)	0.0187 (9)	0.0328 (12)	0.0019 (8)	0.0134 (9)	−0.0012 (7)
C3	0.0331 (11)	0.0229 (9)	0.0415 (13)	−0.0020 (8)	0.0171 (10)	−0.0035 (8)
C4	0.0464 (13)	0.0236 (9)	0.0354 (12)	0.0022 (9)	0.0240 (10)	0.0038 (8)
C5	0.0410 (12)	0.0272 (10)	0.0284 (11)	0.0044 (8)	0.0109 (9)	0.0027 (8)
C6	0.0348 (11)	0.0205 (9)	0.0270 (11)	0.0014 (8)	0.0120 (9)	−0.0018 (7)
C7	0.0339 (12)	0.0314 (10)	0.0312 (12)	−0.0015 (8)	0.0058 (9)	0.0026 (8)
C8	0.0455 (14)	0.0435 (13)	0.0606 (17)	−0.0008 (10)	0.0062 (12)	−0.0204 (11)
C9	0.0364 (13)	0.0465 (13)	0.0544 (16)	0.0002 (10)	0.0106 (11)	−0.0147 (11)
C10	0.0342 (12)	0.0284 (10)	0.0394 (13)	0.0004 (8)	0.0050 (10)	0.0049 (9)
C11	0.0374 (13)	0.0464 (13)	0.0636 (17)	0.0068 (10)	0.0065 (12)	0.0032 (11)
C12	0.0397 (13)	0.0600 (14)	0.0379 (14)	−0.0037 (11)	0.0052 (11)	−0.0007 (11)
C13	0.0361 (11)	0.0271 (10)	0.0305 (11)	−0.0062 (8)	0.0106 (9)	0.0022 (8)

### Geometric parameters (Å, °)

**Table:**

N1—C13	1.331 (2)	C7—H7	1.0000
N1—C1	1.441 (2)	C8—H8A	0.9800
N1—H1	0.8800	C8—H8B	0.9800
O1—C13	1.229 (2)	C8—H8C	0.9800
C1—C6	1.401 (3)	C9—H9A	0.9800
C1—C2	1.402 (3)	C9—H9B	0.9800
C2—C3	1.397 (3)	C9—H9C	0.9800
C2—C10	1.522 (3)	C10—C11	1.529 (3)
C3—C4	1.382 (3)	C10—C12	1.532 (3)
C3—H3	0.9500	C10—H10	1.0000
C4—C5	1.377 (3)	C11—H11A	0.9800
C4—H4	0.9500	C11—H11B	0.9800
C5—C6	1.396 (3)	C11—H11C	0.9800
C5—H5	0.9500	C12—H12A	0.9800
C6—C7	1.520 (3)	C12—H12B	0.9800
C7—C9	1.525 (3)	C12—H12C	0.9800
C7—C8	1.525 (3)	C13—H13	0.9500
			
C13—N1—C1	123.23 (15)	C7—C8—H8C	109.5
C13—N1—H1	118.4	H8A—C8—H8C	109.5
C1—N1—H1	118.4	H8B—C8—H8C	109.5
C6—C1—C2	122.22 (17)	C7—C9—H9A	109.5
C6—C1—N1	119.19 (16)	C7—C9—H9B	109.5
C2—C1—N1	118.57 (17)	H9A—C9—H9B	109.5
C3—C2—C1	117.78 (18)	C7—C9—H9C	109.5
C3—C2—C10	121.42 (17)	H9A—C9—H9C	109.5
C1—C2—C10	120.80 (16)	H9B—C9—H9C	109.5
C4—C3—C2	120.77 (18)	C2—C10—C11	113.87 (18)
C4—C3—H3	119.6	C2—C10—C12	110.56 (16)
C2—C3—H3	119.6	C11—C10—C12	109.77 (18)
C5—C4—C3	120.46 (18)	C2—C10—H10	107.5
C5—C4—H4	119.8	C11—C10—H10	107.5
C3—C4—H4	119.8	C12—C10—H10	107.5
C4—C5—C6	121.17 (19)	C10—C11—H11A	109.5
C4—C5—H5	119.4	C10—C11—H11B	109.5
C6—C5—H5	119.4	H11A—C11—H11B	109.5
C5—C6—C1	117.59 (18)	C10—C11—H11C	109.5
C5—C6—C7	119.45 (17)	H11A—C11—H11C	109.5
C1—C6—C7	122.95 (16)	H11B—C11—H11C	109.5
C6—C7—C9	111.57 (15)	C10—C12—H12A	109.5
C6—C7—C8	111.09 (17)	C10—C12—H12B	109.5
C9—C7—C8	110.50 (18)	H12A—C12—H12B	109.5
C6—C7—H7	107.8	C10—C12—H12C	109.5
C9—C7—H7	107.8	H12A—C12—H12C	109.5
C8—C7—H7	107.8	H12B—C12—H12C	109.5
C7—C8—H8A	109.5	O1—C13—N1	125.92 (17)
C7—C8—H8B	109.5	O1—C13—H13	117.0
H8A—C8—H8B	109.5	N1—C13—H13	117.0
			
C13—N1—C1—C6	−77.0 (2)	N1—C1—C6—C5	−177.80 (15)
C13—N1—C1—C2	104.7 (2)	C2—C1—C6—C7	179.32 (16)
C6—C1—C2—C3	0.1 (3)	N1—C1—C6—C7	1.1 (3)
N1—C1—C2—C3	178.38 (15)	C5—C6—C7—C9	−64.7 (2)
C6—C1—C2—C10	179.73 (16)	C1—C6—C7—C9	116.4 (2)
N1—C1—C2—C10	−2.0 (2)	C5—C6—C7—C8	59.1 (2)
C1—C2—C3—C4	−0.5 (3)	C1—C6—C7—C8	−119.8 (2)
C10—C2—C3—C4	179.90 (17)	C3—C2—C10—C11	−24.2 (3)
C2—C3—C4—C5	0.3 (3)	C1—C2—C10—C11	156.20 (17)
C3—C4—C5—C6	0.3 (3)	C3—C2—C10—C12	99.9 (2)
C4—C5—C6—C1	−0.7 (3)	C1—C2—C10—C12	−79.7 (2)
C4—C5—C6—C7	−179.57 (17)	C1—N1—C13—O1	−2.2 (3)
C2—C1—C6—C5	0.4 (3)		

### Hydrogen-bond geometry (Å, °)

**Table:**

*D*—H···*A*	*D*—H	H···*A*	*D*···*A*	*D*—H···*A*
N1—H1···O1^i^	0.88	2.04	2.910 (2)	171

Symmetry codes: (i) −*x*+1, *y*−1/2, −*z*+1/2.
